# Development of an adult neurofibromatosis clinic in the comprehensive cancer center setting and descriptive analysis of the first 100 patients with neurofibromatosis type 1

**DOI:** 10.1186/s13023-026-04346-x

**Published:** 2026-04-23

**Authors:** Lindsay J. Lipinski, Mary Rose Vater, Ajay Gupta, Jessica Nixon, Jacqueline A. Goldfinch, Shelby Belair, Erika Stewart, Michael Sweeney, Matthew Barth, Kenan Onel

**Affiliations:** 1 Department of Neuro-Oncology, Roswell Park Comprehensive Cancer Center, Buffalo, NY USA; 2 Department of Medicine, Roswell Park Comprehensive Cancer Center, Buffalo, NY USA; 3 Department of Pediatrics, Roswell Park Comprehensive Cancer Center, Buffalo, NY USA; 4 Department of Surgical Oncology, Roswell Park Comprehensive Cancer Center, Buffalo, NY USA; 5 Department of Social Work, Roswell Park Comprehensive Cancer Center, Buffalo, NY USA; 6 Center for Clinical Genomics, Roswell Park Comprehensive Cancer Center, Buffalo, NY USA

**Keywords:** Cancer care facilities, Intersectoral collaboration, Neurofibromatoses, Transition to adult care, Healthcare clinics, Chronic disease

## Abstract

**Background:**

Neurofibromatosis type 1 is a multisystem genetic disorder that most commonly presents with dermatologic manifestations, while also involving the central and peripheral nervous systems. Additional features may include orthopedic, ophthalmologic, and cardiovascular abnormalities, and the condition is further associated with an increased risk of malignancy. Typically, patients receive multidisciplinary care in the setting of a large tertiary-care academic institution within the pediatric department. Here, we describe the development of an adult program housed within a National Cancer Institute-designated comprehensive cancer center and outline our care framework.

**Methods:**

We describe the establishment an adult neurofibromatosis clinic at Roswell Park Comprehensive Cancer Center (Buffalo, NY, USA), including considerations of specialty involvement and expertise, cancer screening and surveillance practices, and clinic workflow. Prospective data were collected and analyzed for the first 100 patients enrolled (2021–2024), including information about their neurofibromatosis-related conditions and cancer history.

**Results:**

Neurofibromatosis-related clinical features in this cohort were consistent with those reported in larger published studies. However, the prevalence of neurofibromatosis type 1-associated malignancy was higher, with 37 diagnoses among 100 patients. This likely reflects the cancer-focused institutional setting of the clinic.

**Conclusions:**

Multidisciplinary neurofibromatosis clinics are essential to address the complex needs of adults with neurofibromatosis type 1. Our experience suggests that a comprehensive cancer center provides an optimal setting; it offers relevant clinical expertise and seamless transition from surveillance to active oncology care for patients who develop malignancy, the most common life-threatening complication in the adult population. We present this model to encourage the development of similar programs and to advance adult neurofibromatosis care with the ultimate goal of improving outcomes and quality of life in this patient population.

.

## Introduction

Neurofibromatosis type 1 (NF1) is an autosomal dominant genetic condition that imposes significant medical, psychological, and social burdens on impacted patients and their families. The condition results from a pathogenic variation in the *NF1* gene, causing functional loss of the protein neurofibromin. This protein is a tumor suppressor, as it regulates cell growth and proliferation through the Ras/MAPK and PI3K/mTOR signaling pathway [1]. Multiple organ systems are impacted, including the nervous system, integumentary system, skeletal system, visual system, and cardiovascular system. The clinical manifestations vary widely, with poor genotype–phenotype correlation [2]. The primary manifestation of NF1 is the development of tumors (both benign and malignant). NF1 is most commonly diagnosed in childhood through clinical manifestations present at 6–10 years of age [3, 4]. As a result, pediatric care has historically been the primary focus of medical attention in the NF1 population. However, adult care is equally important, as NF1 requires lifelong follow-up and the clinical needs of pediatric patients differ substantially from those of adults. These differences underscore that pediatric-focused care alone is insufficient to meet the long-term needs of individuals with NF1 [5, 6].

Adult NF1 patients experience a number of additional medical issues that differentiate their needs from pediatric needs. Such issues include the management of chronic pain, need for family planning and genetic counseling, body image concerns due to the increasing burden of cutaneous neurofibromas, social issues including job stability and need for financial supports for independent living, screening for renovascular hypertension, screening for depression, and the need for additional cancer screening [7–11]. Adult patients may therefore benefit from adult specialist care from providers experienced with NF1 [12, 13]. This has been demonstrated by the change in management for the vast majority of adult patients referred to an NF1 specialty center upon establishing care [12]. Early recognition and treatment of these “adult-specific” NF1-related health issues have the potential to save lives and preserve quality of life.

Because the spectrum of clinical manifestations in patients with NF1 is diverse, care is ideally provided by a multidisciplinary team of subspecialists with expertise in this condition. This is evidenced by the nationally increasing number of NF1 comprehensive specialty care centers, but most are focused on the care of pediatric patients [14, 15]. Based on 2018 data, pediatric patients in the U.S. would have to travel a median of 67.9 miles (109 km) to reach a certified clinic for their age group, while adult patients would have to drive a median of 295.8 miles (476 km) [13]. The Neurofibromatosis Clinic Network (NFCN), amongst other advocacy groups, supports a multidisciplinary approach to care to “standardize and raise the level of neurofibromatosis clinical care nationally [16–20].” Goals of a comprehensive clinic include providing longitudinal care, determining treatment paths, monitoring and managing this complex disease, and educating and supporting the patient and family [10, 11, 17, 21].

The majority of established NF clinics are run by pediatric-trained providers and are located within pediatric hospitals [12]. There are a limited number of adult-focused clinics with adult-trained providers [13]. Without a comprehensive adult clinic, care of NF patients often becomes fragmented upon reaching adulthood. Care then takes place across a number of medical and surgical specialists in the community who may lack in-depth training related to the condition [4, 12, 15, 22]. Adults with the condition may feel uncomfortable or unwelcome at a pediatric facility or do not receive medical care altogether due to geographic or financial limitations. In the absence of an adult program in our region, it was apparent that patients were being seen in the pediatric setting, travelling elsewhere, or receiving no specialized NF1 care at all despite the clear impact on the disease-related health and well-being of these patients.

While often mistaken as a dermatologic condition, NF1 is a cancer predisposition syndrome. High-grade malignancies associated with NF1 include malignant peripheral nerve sheath tumor (MPNST), breast cancer, and high-grade glioma [7, 11]. These patients have an overall cancer risk that is 5 to 15% higher than that in the non-NF1 population, with a lifetime risk of 60% [23]. Additionally, patients with NF1 are noted to have earlier onset of cancer, worse overall prognosis, and lower survival [12, 24]. The increased cancer risk and its influence on morbidity and mortality mandates systematic medical follow-up. A cancer center with specialists in tumor pathophysiology, ongoing research, and innovative clinical care is an ideal home for the development of a comprehensive program [10, 25].

## Methods

### Development of the clinic

The clinic was established within Roswell Park Comprehensive Cancer Center, a National Cancer Institute-designated comprehensive cancer center in Buffalo, New York, USA. Roswell Park is a 157-bed tertiary academic center serving an urban, suburban and large rural catchment area of approximately 1.5 million people. As access to care in the U.S. largely depends on insurance, patients’ ability to establish care was influenced by the insurance status; however, the institution accepts most private and public plans and offers financial assistance for uninsured and underinsured patients.

Given the multisystem manifestations of NF1, the clinic recruited specialists in neurology/neuro-oncology, neurosurgery, internal medicine/medical oncology, surgical oncology, plastic surgery, pain management, medical genetics/genetic counseling, and social work. Additional referral pathways were created with high-risk breast cancer screening (internal), neuropsychology (external), and ophthalmology (external). These specialties and resources are commonly part of the comprehensive cancer center setting and therefore available for collaboration within the NF1 clinic framework. A dedicated nurse coordinator was appointed once the multidisciplinary team was assembled.

Because many patients were transitioning from a long-established pediatric program, we partnered with them to implement a formal transition plan. Pediatric providers introduced the concept of transition in late adolescence and facilitated a “warm handoff” to explain the role and importance of adult-specific care. While no specific age criterion was set, the pediatric clinic employed the Six Core Elements of Transition™ as a framework (Table 1) [22, 26]. This partnership is a key component of the development of our program and avoids gaps in care that can be problematic in other settings [18].

**Table 1:** Six Core Elements of Health Care Transition™ (reproduced with permission from the authors) [26]

Step	Element	Explanation	Age Range
1	Policy/Guide	Develop, discuss, and share transition and care policy/guide	12–14
2	Tracking & Monitoring	Track progress using a flow sheet registry	14–18
3	Readiness	Assess self-care skills and offer education on identified needs	14–18
4	Planning	Develop transition plan with medical summary	14–18
5	Transfer of Care	Transfer to adult-centered care and to an adult practice	18–21
6	Transition Completion	Confirm transfer completion and elicit consumer feedback	18–23

**Fig. 1:**
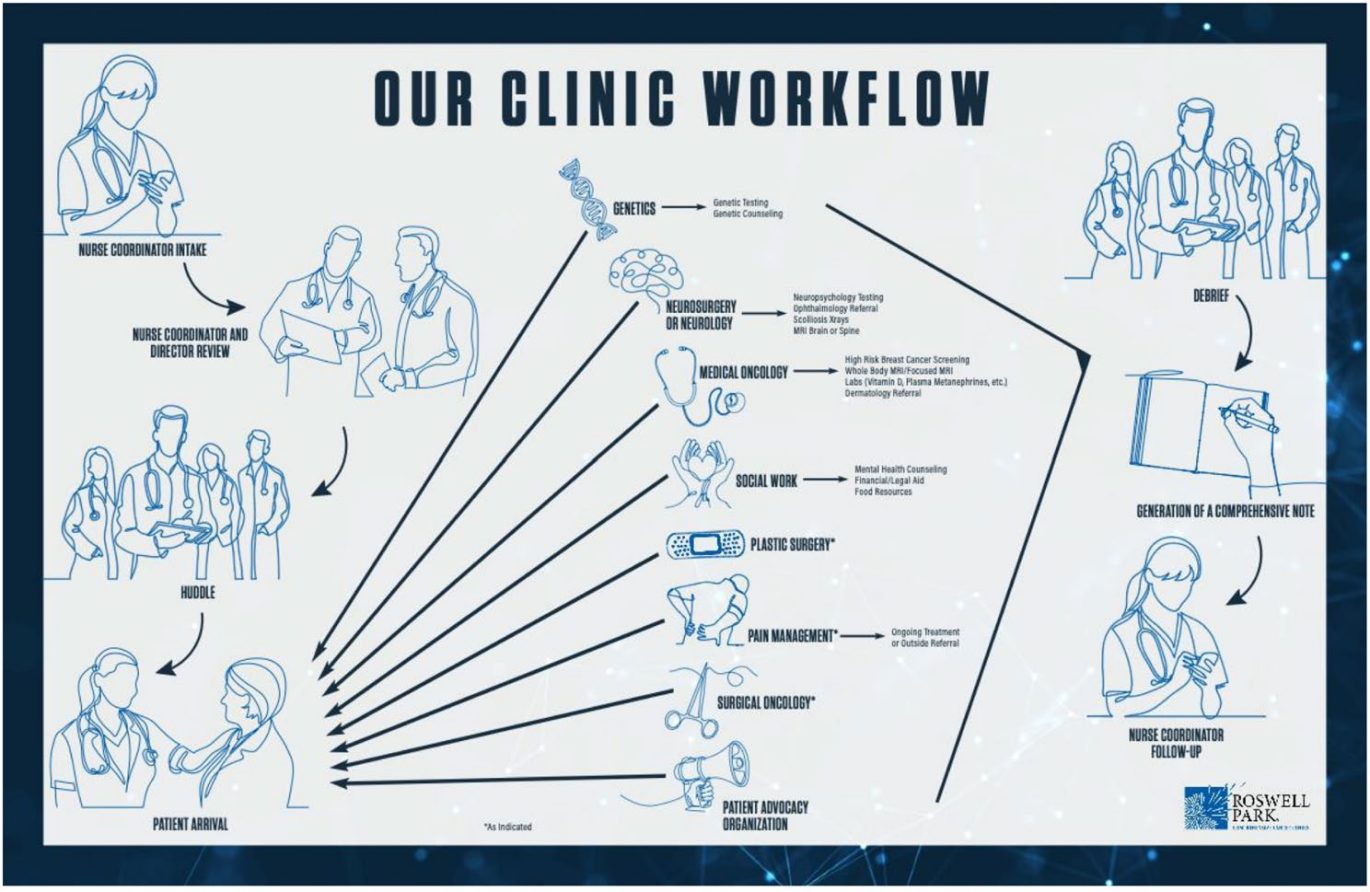
Customized workflow for the adult neurofibromatosis type 1 (NF1) clinic

Utilizing the shared resources of established clinics within the cancer center and provider experience as a basis, a customized workflow was created (Fig. 1). Before the first visit, the nurse coordinator conducted an extensive phone intake. Patients with uncertain diagnoses were evaluated before being formally enrolled. Care plans were individualized but aligned with expert consensus and published guidelines [11, 26].

Initial clinic promotion occurred internally through emails, intranet postings, educational pamphlets, and staff announcements. An external webpage was created to streamline referrals and provide patient resources. Outreach expanded to community mailers and community physician education sessions. Approximately 10 educational events occurred in year 1, and 3–5 annually thereafter. Collaboration with the regional patient advocacy organization offered bidirectional benefits, including access to resources and expanded patient engagement.

Before each clinic session, the director and nurse coordinator reviewed all patient information. A pre-clinic huddle set preliminary care plans, and a post-clinic debrief consolidated next steps. At the initial visit, patients were oriented to the clinic and provided with resources and contacts. Patients remained in a single exam room while all providers rotated to them. Patients were evaluated by a neurologic specialty (neuro-oncology or neurosurgery) and medical and/or surgical oncology at all visits, with additional providers as needed. The social worker saw all patients at their first visit to screen for mental health needs and assess for community resource needs. A comprehensive medical, neurologic, and dermatologic exam was performed, including blood pressure screening and evaluation for scoliosis and neurocognitive concerns. Whole body MRI was offered to patients at entry to screening to assess burden of plexiform neurofibromas, with targeted body imaging obtained annually thereafter in locations of identified plexiform neurofibromas (e.g. brachial plexus, pelvis, etc.) [27]. A baseline MRI of the brain was offered if a patient had never had imaging before, but other neurologic imaging was obtained only for patient symptoms or exam findings. Pain management, genetic testing, and laboratory evaluation (e.g., plasma free metanephrines, vitamin D) were pursued as indicated. Patients with optic pathway gliomas or no prior brain imaging were encouraged to undergo annual or biannual ophthalmologic evaluation due to the low but documented risk of late-onset visual decline [28, 29]. Eligible women were referred for high-risk breast cancer screening in accordance with National Comprehensive Cancer Network (NCCN) guidelines [17, 20]. Referrals for plastic surgery were made when appropriate.

Annual follow-up included a brief pre-visit phone intake, neurologic and general medical evaluations, indicated imaging and/or labs, and specialty visits based on ongoing needs. Clinic sessions initially occurred every 3 months but increased to every 6 weeks as the patient volume grew, with further expansion anticipated. After each visit, all subspecialists documented in the medical record, and a comprehensive summary note was generated to be sent to the patient’s primary care provider and other involved clinicians.

### Patient analysis

At the time of first visit to the NF1 clinic, patients were prospectively enrolled into an IRB approved registry to track their demographics, NF1-related conditions and diagnoses, and cancer history. This database was also used clinically to track screening and surveillance adherence, recommended intervals for imaging studies, outside referrals, and NF1-related treatments. Once data collection of the first 100 patients was complete, a basic analysis was performed and compared against published literature of larger patient populations.

## Results

**Fig. 2:**
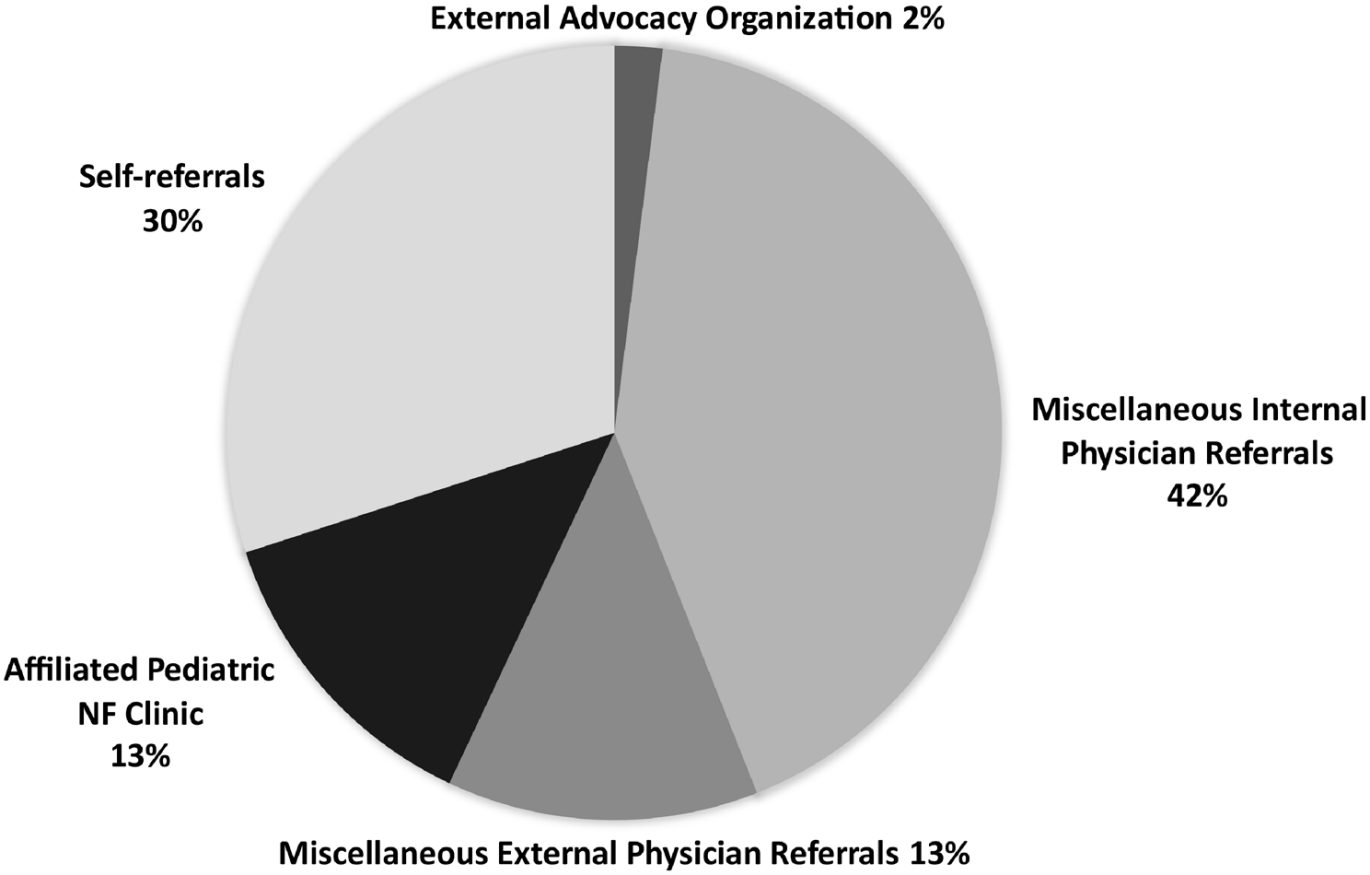
Sources of referrals to the NF1 clinic

Data were collected over a four-year period from 2021 to 2024. Participants were followed for a median of 27.6 months (IQR: 15.2–39.6), with a mean follow-up of 26.5 months (SD: 13.8). Median age at the time of presentation was 39.5 years old (range 21 to 75). Fifty-five patients were female; 45 were male. Only 22 patients received prior pediatric care; most patients were receiving NF1-specific care for the first time with enrollment into the clinic. Referrals to the clinic remained relatively stable over the 4 years, within a range of 21 to 29 patients. Four patients died during the data collection period, 2 from malignant peripheral nerve sheath tumors (MPNST), 1 from cerebellar anaplastic astrocytoma, and 1 from presumably unrelated Creutzfeldt-Jacob disease. Three patients elected to no longer follow-up with the clinic due to relocation, and 3 could not be reached after 1 or 2 clinic visits. Referral sources were varied (Fig. 2); at the time of analysis, the majority of patients came from internal referrals within the cancer center, reflecting the initial internal messaging about the creation of the clinic.

Among internal referrals, specialty types included surgical oncology (*n* = 19), medical oncology (*n* = 9), neurosurgery (*n* = 9), neurology (*n* = 3), genetics (*n* = 3), and the outreach clinic (*n* = 1). Of note, no referrals were received from the dermatology clinic, which typically does not treat benign skin conditions in the cancer center setting. Among external referrals, specialty types included primary care (*n* = 5), neurosurgery (*n* = 4), ophthalmology (*n* = 1), neurology (*n* = 1), obstetrics and gynecology (*n* = 1), and pediatrics (*n* = 1). Self-referred patients cited family members (including adults whose children were enrolled in the pediatric clinic), the institutional website, or another online resource such as the Neurofibromatosis Clinic Network as a means of identifying the clinic. Referrals from the adult clinic were also made for children of adult patients who were not receiving care or young children who were recently diagnosed.

Forty-one patients had a family history of NF1, with 54 having no known family history and 5 patients without knowledge of their family history due to adoption. Sixty-one patients completed genetic testing; of those, a pathogenic variant was not identified in only 4 (6.5%) patients, all of whom met clinical diagnostic criteria and who were suspected to be mosaic or segmental, consistent with reported rates of pathogenic variant identification in NF1 [30, 31]. The remaining patients are in the process of genetic testing (*n* = 12) or declined to undergo testing (*n* = 27). Patients who declined genetic testing most commonly cited lack of perceived impact on clinical management or family planning decisions, rather than encountering obstacles related to insurance coverage or cost.

In regard to clinical manifestations of NF1, see Table 2 for statistics on common features in relation to historical data. For those listed in the unknown category, patients either declined to have imaging performed at intake (e.g. MRI of brain to assess presence of optic pathway glioma, whole body MRI to assess presence of plexiform neurofibroma), or the specific finding was not documented in the patient’s record (e.g. Lisch nodules, axillary freckling). Amongst the patients with known plexiform neurofibromas, 8 patients had undergone treatment or are currently being treated with a MEK inhibitor, which targets mitogen-activated protein kinases. Discontinuation of treatment was based on intolerable side effects. Treatment has been continued in patients who are tolerating the therapy and have no demonstrated disease progression. Our clinic plans to continue this treatment as long as this remains the case, awaiting further studies on the optimal length of therapy with this class of drugs in NF1. One additional patient was treated with a MEK inhibitor for a progressive, symptomatic optic pathway glioma.

**Table 2:** Presence or absence of clinical features associated with NF1 out of a total of 100 enrolled patients

Clinical Manifestation	Present	Absent	Unknown	Historical Prevalence
Café au lait spots	94%	5%	1%	91–95% [2, 4]
Axillary freckling	67%	32%	1%	84–86% [2, 4]
Lisch nodules	27%	68%	5%	57–66% [2, 4]
Cutaneous neurofibromas	93%	7%	0%	90–99% [9, 32]
Plexiform neurofibromas	44%	43%	13%	24–50% [2, 33]
Optic pathway glioma	16%	76%	8%	15–20% [34]
Scoliosis	28%	71%	1%	10–30% [35]

* Total patient number analyzed=100; percent presented equal to n

Within our patient population, there were 45 diagnoses of malignancy among 35 patients over the patients’ lifetimes through the time of data collection. Nine patients had more than one primary tumor, all metachronous, in line with other known larger studies [36]. Of the malignancy diagnoses, 37 (82.2%) were considered to have a known association to NF1 (see Table 3). The overall prevalence of MPNST was 9%, though 20% among patients with known plexiform neurofibromas. Additional tumor types with uncertain or unlikely relationships to NF1 included atypical meningioma (*n* = 2), basal cell carcinoma of the head (*n* = 1), squamous cell carcinoma of the tonsil (*n* = 1), cervical carcinoma (*n* = 1), multiple myeloma (*n* = 1), adenocarcinoma of the small intestine (*n* = 1), and thyroid carcinoma (*n* = 1) [36]. Seven of the NF1-associated malignancies were diagnosed as a direct result of enrollment into the clinic: primary brain tumors (*n* = 3), breast cancer (*n* = 2), pheochromocytoma (*n* = 1), and MPNST (*n* = 1). Seventy-nine percent of eligible women (*n* = 37) elected to undergo screening through the high-risk breast cancer screening clinic.

**Table 3:** Prevalence of malignancy with known association with NF1 in this patient population

Tumor Type	Study %	Historical %
Breast	12%	2.9–4.7% [7, 24]
MPNST	9%	1–2% [37]
Low-grade glioma (excl. OPG)	6%	5.5% [24]
GIST	5%	5–25% [38]
Lymphoma	2%	0.3% [24]
Pheochromocytoma	2%	0.1–5.7% [16]
High-grade glioma	1%	1.7% [24]

* Total patient number analyzed = 100; percent presented equal to n. OPG, optic pathway glioma

## Discussion

Expanding access to comprehensive care can have numerous positive consequences for patients, their families, and our community [15, 24]. Over a 4-year period, the adult neurofibromatosis clinic accrued 100 unique patients. Several early challenges emerged during clinic development that offer important lessons for similar programs. Recruitment of specialists was the most significant initial barrier, as no providers were allocated protected time for clinic participation. Engagement was strongest among clinicians already caring for NF1 patients or motivated by the unmet needs of this population, underscoring the importance of early identification of invested provider champions. Establishment of a dedicated nurse coordinator with protected time proved critical to clinical functionality and should be considered essential for multidisciplinary care models.

Scheduling a common clinic time across specialties required substantial coordination, and identifying a physical space that allowed for prolonged, multi-provider visits was a logistical challenge. Limited exam room availability constrained patient throughput and remains a growth barrier, highlighting the need to anticipate space requirements early in program planning. During the study period, the clinic operated without dedicated funding beyond nurse coordinator salary support, relying on existing departmental resources. While subsequent funding has since been obtained, early financial limitations emphasize the importance of institutional backing to support sustainability, education, and research.

In combination with the enrollment in the established pediatric clinic, we estimate approximately 43%–51% of individuals with NF1 in the Western New York region are currently captured based on prevalence estimates [39, 40]. Although the regional population is expected to remain stable [41], continued outreach will be necessary to reach a larger proportion of affected adults. While internal referrals initially predominated, reflecting the cancer center setting, this referral source is expected to plateau. As a result, community outreach has become a strategic priority. Messaging to the community has been intentionally framed to complement, rather than replace, existing community-based care, a key consideration for maintaining referral relationships and trust. This philosophy is also reinforced through concise multidisciplinary summary notes that facilitate clear communication with referring providers.

The cancer center environment proved particularly advantageous for adult NF1 care. Chronic pain is the most disabling concern in adults with NF1 and exposes those with NF1 to physical suffering and psychological distress, in turn contributing to overall psychosocial dysfunction [42–45]. Pain in our patient population was effectively addressed through a robust pain management service, as is common in cancer centers. Similarly, consistent access to a social worker, attune to the psychosocial, psychiatric, financial, self-image related, and work-related stressors, proved a valuable resource for adults [46, 47]. Cutaneous neurofibromas can also significantly impact quality of life, particularly those on the face, and intervention may be helpful [9]. Plastic surgery was selectively utilized for painful or disfiguring cutaneous neurofibromas. A substantially higher number of eligible women underwent high-risk breast cancer screening than other published community-based data [12], and patients also benefited from high-quality, consistent, routine imaging studies reviewed by experienced radiologists with expertise in cancer detection and familiarity with NF1. These services, often difficult to coordinate across fragmented systems, were readily accessible within the cancer center infrastructure.

Analysis of this cohort demonstrated clinical manifestations largely consistent with prior reports, apart from the higher observed prevalence of malignancy. This finding likely reflects referral bias inherent to a tertiary cancer center, as many patients initially presented for cancer-related care. The malignancy prevalence of 35% exceeds that reported in larger cohorts [17, 36]. Current literature suggests a lifetime risk of 8–15.8% of patients with NF1 develop MPNST [23, 37, 48], while our patient population had a 9% prevalence in the short follow-up period. This likely reflects referrals to a tertiary care center with expertise in sarcoma treatment, a bias which has been previously described [37]. Similarly, breast carcinoma, glioma, gastrointestinal stromal tumor, lymphoma, and pheochromocytoma prevalence are higher than reported by a large comprehensive cancer center and in other larger cohort studies [2, 19, 24]. Most notable is the prevalence of breast carcinoma in our population, with 12% being significantly higher than previously reported 2.9–4.7% [7, 24]. This likely reflects the small sample size of this population within a single institution, which is a significant limitation in the reporting of these statistics. These biases highlight the importance of contextualizing early outcome data and avoiding overinterpretation of prevalence estimates during initial clinic development.

Despite variability across NF1 clinics nationally and internationally, cancer centers encompass many specialties necessary for adult care in patients with this tumor predisposition syndrome. This framework also provides an easy transition from surveillance to active cancer care [16, 23]. The identification of multiple malignancies in the short time that our clinic has been established supports this model and reinforces the importance of provider familiarity with NF1-related cancer risk and symptom-driven screening guidelines [27, 49]. As the clinic continues to expand, anticipated challenges include increasing demand for space, protected provider time, and sustained funding. Addressing these needs will be essential to maintaining high-quality care and extending the benefits of multidisciplinary, adult-focused NF1 management.

## Conclusion

NF1 is a chronic, multisystem tumor predisposition syndrome that requires lifelong, coordinated, multidisciplinary care extending beyond childhood. Our experience demonstrates that an adult multidisciplinary clinic embedded within a comprehensive cancer center is an effective model for addressing the complex medical and oncologic needs of this population. This setting offers specialized expertise, integrated screening, and the ability to transition seamlessly from surveillance to active cancer care.

Among the first 100 patients enrolled into our clinic, clinical manifestations were consistent with published cohorts, while the higher observed burden of malignancy reflects the medical setting reinforces the importance of systematic follow-up in an appropriately resourced setting. Importantly, the successful implementation of this clinic highlights several key lessons for program development, including the necessity of dedicated care coordination, early identification of clinician champions, proactive planning for space and staffing, intentional community outreach, and structured pediatric-to-adult transition. Together, these insights underscore the value of specialized adult NF1 programs and support broader adoption of similar models to improve access, outcomes, and quality of life for adults with NF1.

## Data Availability

The datasets generated and analyzed during the current study are not publicly available because they contain protected health information. Anonymized data are available from the corresponding author on reasonable request.
